# Genomic and functional conservation of lncRNAs: lessons from flies

**DOI:** 10.1007/s00335-021-09939-4

**Published:** 2022-01-31

**Authors:** Carlos Camilleri-Robles, Raziel Amador, Cecilia C. Klein, Roderic Guigó, Montserrat Corominas, Marina Ruiz-Romero

**Affiliations:** 1 Departament de Genètica, Microbiologia I Estadística, Facultat de Biologia and Institut de Biomedicina (IBUB), Universitat de Barcelona, Barcelona, Catalonia Spain; 2 Centre for Genomic Regulation (CRG), The Barcelona Institute for Science and Technology (BIST), Barcelona, Catalonia Spain; 3 Universitat Pompeu Fabra (UPF), Barcelona, Catalonia Spain

**Keywords:** LncRNAs, *Drosophila melanogaster*, Flies, Conservation, Comparative genomics, Development

## Abstract

Over the last decade, the increasing interest in long non-coding RNAs (lncRNAs) has led to the discovery of these transcripts in multiple organisms. LncRNAs tend to be specifically, and often lowly, expressed in certain tissues, cell types and biological contexts. Although lncRNAs participate in the regulation of a wide variety of biological processes, including development and disease, most of their functions and mechanisms of action remain unknown. Poor conservation of the DNA sequences encoding for these transcripts makes the identification of lncRNAs orthologues among different species very challenging, especially between evolutionarily distant species such as flies and humans or mice. However, the functions of lncRNAs are unexpectedly preserved among different species supporting the idea that conservation occurs beyond DNA sequences and reinforcing the potential of characterising lncRNAs in animal models. In this review, we describe the features and roles of lncRNAs in the fruit fly *Drosophila melanogaster,* focusing on genomic and functional comparisons with human and mouse lncRNAs. We also discuss the current state of advances and limitations in the study of lncRNA conservation and future perspectives.

## Introduction

Long non-coding RNAs (lncRNAs), are DNA sequences encoding transcripts larger than 200 nt that lack protein-coding potential. Although many lncRNAs show low levels of expression, some are known to play a pivotal role in the regulation of several cellular processes. In recent years, the amount of available transcriptomic data has increased exponentially and has been crucial in demonstrating that genomes are extensively transcribed. Additionally, the emergence of tools to identify putative non-coding genes has led to the annotation of a large number of lncRNAs not only in humans, but also in mice, insects and plants (Brown et al. 2014; Derrien et al. 2012; Lagarde et al. 2017; Legeai and Derrien 2015; Paytuví Gallart et al. 2016; Pervouchine et al. 2015).

The most updated version of the human genome annotation contains 19,951 protein-coding genes and 17,948 lncRNA genes (GENCODE v37, March 2021). In contrast to protein-coding genes, whose molecular functions can often be inferred by the presence of protein domains, inferring the function, if any, of lncRNAs is a whole different story. Although some lncRNAs have been functionally characterised in humans (Rinn et al. 2007; Tripathi et al. 2010; Wutz et al. 2002; Zhou et al. 2007), the frequent absence of phenotypes after their mutation or deletion has raised questions about the proportion of annotated lncRNAs that are actually functional (Gao et al. 2020; Lee et al. 2019).

High-throughput screens of lncRNA knock downs affecting molecular phenotypes have been performed in human cells (Liu et al. 2017, 2018; Ramilowski et al. 2020). However, the difficulties in conducting functional genetic screens in humans and other vertebrates in vivo limit the ability to characterise the role of annotated lncRNAs in these species, pointing out the need to use less complex model organisms. One of the most useful animal models for genetic analyses is the fruit fly *Drosophila melanogaster*, whose genome contains 13,969 protein-coding and 2545 long non-coding RNA annotated genes (FlyBase r6.39, February 2021). A huge advantage of using *Drosophila* as an animal model is the availability of a great variety of genetic tools, resources and mutant collections that facilitate the undertaking of genetic screens. Forward genetic screens use mutagenesis to create random mutations in the search for the genotypes that underlie the resulting phenotypes. They have been instrumental in identifying the function of protein-coding genes (St Johnston 2002). On the contrary, reverse genetic assays are preferentially used to screen for lncRNAs, searching for phenotypes after creating targeted mutations in candidate genes (Wen et al. 2016). Compared to protein-coding genes, in which a single nucleotide deletion or insertion can abolish the production of the proteins they encode, the deletion of a large region encompassing the whole gene, smaller specific domains or the promoter, may be required to compromise the function of lncRNAs. The existence of different systems for the conditional expression of transgenes (both for inhibition and/or overexpression), widely used to assess protein-coding genes, may also contribute to understanding the function of lncRNAs.

It is well known that fundamental biological mechanisms and signalling pathways are conserved throughout evolution. An estimated 75% of genes related to human diseases have orthologs in the *Drosophila* genome (Bier 2005; Ji et al. 2019), endorsing the study of human diseases in flies. In this context, several lncRNAs have been associated with cancer (Dong et al. 2015; Li et al. 2014, 2015; Wu et al. 2014), and many neurological disorders, such as amyotrophic lateral sclerosis (Zu et al. 2013), Alzheimer’s disease (Lee et al. 2015) and Huntington’s disease (Johnson 2012). Although no fly orthologs have been identified for lncRNAs associated to human diseases, the study of lncRNAs in *Drosophila* could shed light into the regulation of disease-causing genes (Li et al. 2012; Lo Piccolo and Yamaguchi 2017; reviewed in Rogoyski et al. 2017).

In this review, we discuss lncRNAs in the fly genome and compare them with human and mouse lncRNAs. Furthermore, we provide an overview of the functions and mechanisms of action associated with lncRNAs in *Drosophila*, including similarities in the function of some lncRNAs between flies and humans. We also characterise developmentally dynamic fly lncRNAs that are differentially expressed during tissue development, and report resemblances among these lncRNAs and the ones identified in human and mouse organ development (Sarropoulos et al. 2019). Finally, we discuss the current status of identifying orthologues in evolutionarily distant species such as flies and humans.

## Genomic and transcriptomic comparison between flies, mice and humans

**Fig. 1:**
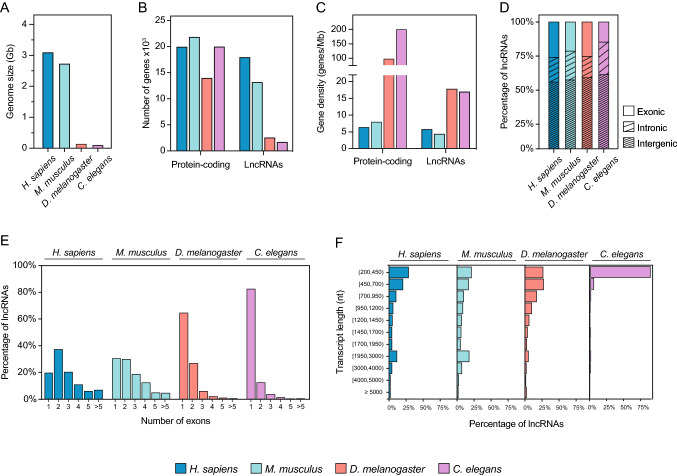
Genomic and transcriptomic comparison of humans, mice and flies. **a** Barplot showing the size (gigabases—Gb) of the human (*H. sapiens*), mouse (*M. musculus*), fly (*D. melanogaster*) and worm (*C.elegans*) genome. **b** Number of annotated protein-coding and long non-coding RNA (lncRNA) genes in each species. **c** Gene density, measured as the number of genes per megabase, of protein-coding and lncRNA genes in each species. **d** Classification of the annotated lncRNA genes into exonic, intronic or intergenic groups. The longest annotated isoform of each lncRNA has been used for overlap analysis and classification. **e** Distribution of the lncRNA genes annotated in human, mouse, fly and worm depending on their number of exons. **f** Distribution of the long non-coding genes of humans, mice, flies and worms based on the size (nucleotides—nt) of their longest transcript. Human data from GENCODE v37 are shown in blue, mouse data from GENCODE M27 are shown in cyan, fly data from FlyBase r6.39 are presented in red and worm data from WS281 are shown in pink

*Drosophila* has four pairs of chromosomes: one pair of sexual chromosomes and three pairs of autosomes. In flies, similar to what happens in mice and humans, sex is determined by the XX/XY mechanism, with females carrying two X chromosomes and males carrying one X and Y chromosomes. In mammals, the presence of the Y chromosome determines the male sex, while its absence results in female individuals. However, the Y chromosome is not involved in sex determination in flies. Instead, the X:A ratio is responsible for the activation of the feminizing gene Sex-lethal (sxl). Hence, flies carrying XY or X0 are male, while flies carrying XX or XXY are female. The *Drosophila* genome is small, with approximately 120 megabases, (Adams et al. 2000) compared to the human and mouse genomes (3100 and 2700 megabases, respectively) (Lander et al. 2001; Venter et al. 2001; Consortium et al. 2002) (Fig. 1a). This is consistent with the reduced number of annotated genes in *Drosophila* (17,874) compared to humans and mice (45,468 and 39,923 genes, respectively). This trend is preserved for both protein-coding genes (13.969 genes in flies compared to 19,951 and 21,848 genes in humans and mice, respectively) and lncRNA genes (2545 genes in *Drosophila* compared to 17,948 and 13,186 genes in humans and mice, respectively) (Fig. 1b). Remarkably, the number of lncRNAs in *Drosophila* is considerably smaller compared to protein-coding genes, whereas in mice and humans the number of lncRNAs and protein coding-genes is similar (Fig. 1b). Furthermore, the *Drosophila* genome is much more compact, containing around 100 protein-coding and 18 lncRNA annotated genes per megabase compared to fewer than 10 protein-coding and lnRNA genes per megabase in the human and mouse genomes (Fig. 1c).

LncRNAs are pervasively distributed throughout the genome and can be found in intergenic regions (lincRNAs) or overlapping totally or partially with sequences of other genes transcribed in the same direction (sense) or in the opposite direction (antisense). Despite the differences in genome compactness among *Drosophila*, mice and humans, lincRNAs represent 50–55% of all annotated lncRNAs in the three species. Similarly, the proportion of lncRNAs found overlapping the introns (intronic) or exons (exonic) of other genes accounts for ~ 20% and ~ 25%, respectively, in all three species (Fig. 1d). Regarding the number of exons, the majority of lncRNAs found in *Drosophila* are either mono-exonic or composed of 2 exons, with only a few exceptions containing 3 or more exons. On the contrary, the number of exons in human and mouse lncRNAs is more diverse, with around 60% in each species containing 1–2 exons (compared to 90% in fly) and around 20% containing 4 or more exons (Fig. 1e). This is consistent with the proportion observed in protein-coding genes in flies (Graveley et al. 2011). In terms of transcript size, as with the protein-coding genes, *Drosophila* lncRNAs are shorter than human and mouse lncRNAs (average length of 962 nt in flies compared to 1230 and 1456 nt in humans and mice, respectively, Mann–Whitney–Wilcoxon test *p*-value < 1e−12 for all comparisons). Only 3.35% of *Drosophila* lncRNAs span more than 3 kb compared to 7.78% in humans and 10.35% in mice (Fig. 1f).

The small number of lncRNA genes annotated in *Drosophila*, with respect to humans and mice may not be a biological feature, but it might rather reflect two factors that hinder the identification of lncRNAs in *Drosophila*. First, the properties of most lncRNAs (low expression and high specificity in terms of time and tissue) might constraint their expression to a specific region during a very specific period of time. Thus, the identification of these lncRNAs may require transcriptomic analyses in specific tissues and developmental stages, which are not as common in flies as they are in humans and mice. Supporting this hypothesis, a recent publication producing transcriptomic data from *Drosophila* embryonic mesodermal cells collected at different developmental stages identified 179 novel lncRNA genes that could play a role in embryogenesis (Schor et al. 2018). Second, the bias towards mono-exonic genes found so far in *Drosophila* could also affect the identification of novel lncRNA genes, since most identifying pipelines often omit novel mono-exonic transcripts in favour of spliced, multi-exonic transcripts to reduce false positives. To provide a global vision of the evolutionary trend we observed for lncRNAs in flies, we inspected the genome of the worm *Caenorhabditis elegans* (Fig. 1). Interestingly, the genomic features we interrogated indicate that lncRNAs in worms resemble fly lncRNAs compared to human and mouse lncRNAs.

## Functions and mechanisms of action of *Drosophila* lncRNAs

Since the discovery of lncRNAs, several studies in mammals and flies have demonstrated that lncRNAs participate in a variety of cellular processes, such as development, differentiation and proliferation, and often contribute to the modulation of gene expression programmes (reviewed in Jandura and Krause 2017; Statello et al. 2021). Functional lncRNAs can be classified as *cis*-acting lncRNAs, when they act near their site of transcription within the same molecule, or *trans*-acting lncRNAs, which act far from their locus or in a different DNA molecule. In general, lncRNAs influence gene expression at three main levels: chromatin regulation, transcriptional regulation and post-transcriptional regulation (reviewed in Statello et al. 2021). On chromatin, some lncRNAs regulate the activity or localisation of chromatin regulatory complexes and transcription factors. These lncRNAs bind to specific chromatin regions and interact with proteins, facilitating or inhibiting their binding to targeted neighbouring genes, thereby promoting or repressing gene expression (Grote and Herrmann 2013; Jiang et al. 2015; Prensner et al. 2013; Rinn et al. 2007; Yap et al. 2010). At the transcriptional level, lncRNAs influence gene regulation directly by interacting with the transcriptional machinery, mediating or facilitating loops between promoters and enhancers (eRNAs) or, in some cases, the act of transcription or splicing of a lncRNA influence the transcription of nearby genes (reviewed in Statello et al. 2021). LncRNAs can also act at the post-transcriptional level by interacting with a plethora of RNA-binding proteins that contribute to mRNA stability, localisation, splicing or translation (Cao et al. 2017; Gumireddy et al. 2013; Lee et al. 2016; reviewed in He et al. 2019).

In the last few years, several lncRNAs have been characterised in *Drosophila*. Although most of them are not evolutionarily conserved across metazoans at the sequence level, some seem to participate in similar cellular processes as those in mammals, such as dosage compensation or Polycomb group (PcG)/Trithorax group (TrxG) regulation (reviewed in Murillo-Maldonado and Riesgo-Escovar 2019; Ringrose 2017; Samata and Akhtar 2018). In this section, we discuss the functions associated with fly lncRNAs, their level of conservation in mammals, and provide some specific examples.

### LncRNAs influencing chromatin regulation

#### LncRNAs involved in dosage compensation mechanisms

As mentioned before, in *Drosophila*, sex is dictated by the XY sex-determination system. Comparable to that occurring in mammals, the imbalance in the expression of X-linked genes between females and males is corrected by a dosage compensation mechanism, involving lncRNAs, which result in similar levels of expression of the genes in the X chromosome. However, in female mammals, one of the X chromosomes is subjected to inactivation, whereas in *Drosophila*, the transcription rate of the male X chromosome is almost doubled. These strategies share some mechanistic similarities, including the involvement of lncRNAs. In both cases, a lncRNA is responsible for recruiting chromatin-modifying complexes that drive the inactivation (in female mammals) or overactivation (in male flies) of the X chromosome. Briefly, in mammals, the lncRNA *Xist* is upregulated in one of the X chromosomes of the females at early embryonic stages and rapidly spreads along the X chromosome from which it is transcribed (Brockdorff et al. 1992; Brown et al. 1991, 1992). Polycomb repressive complex 2 (PRC2), a chromatin regulatory complex, is recruited by *Xist* and mediates the trimethylation of lysine 27 in the histone H3 tail (H3K27me3). This triggers the heterochromatinisation of the *Xist*-bound X chromosome, resulting in X chromosome inactivation (Lee et al. 1996; Penny et al. 1996; Wutz and Jaenisch 2000).

On the contrary, in flies, the male-specific lethal complex (MSL), composed of MSL proteins and the lncRNAs *roX1* and *roX2*, is responsible for the overactivation of genes located in the X chromosome of *Drosophila* males. Although very different in size and sequence, *roX1* and *roX2* act redundantly to allow the binding of MSL2 and the other subunits of the complex, which target the X-chromosome in males (Meller and Rattner, 2002). The MSL subunits mediate the activation of the X-chromosome genes by the acetylation of lysine 16 in histone H4 (H4K16ac) (Bone et al. 1994; Gelbart et al. 2009). In female flies, the *Sex lethal* (*sxl*) gene, is upregulated and the female-specific RNA-binding protein it encodes interacts with the *msl2* mRNA to inhibit its translation, preventing the assembly of the MSL complex and the subsequent dosage compensation (Beckmann et al. 2005; Gebauer et al. 1998; Graindorge et al. 2013).

#### LncRNAs mediating PcG and TrxG gene regulation

PcG and TrxG proteins are key modulators of an evolutionarily conserved gene regulatory system. They are chromatin modifiers that operate antagonistically and were originally identified as part of an epigenetic cellular memory system that maintains repressed or active gene expression states. The first identified target genes of PcG and TrxG regulation were the fly Hox genes (reviewed in Kassis et al. 2017). Hox genes encode transcription factors that determine the allocation of segmental identity along the anterior–posterior body axis and when mutated, typically, lead to homeotic transformations (reviewed in Mallo and Alonso 2013). In *Drosophila*, Hox genes are organized in two separate gene clusters: the Antennapedia and Bithorax complexes (ANT-C and BX-C, respectively); and their expression is activated by the segmentation gene products in early fly development. Further characterisation of Hox loci allowed the identification of several elements that respond to PcG and TrxG genes, named Polycomb response elements (PREs) and Trithorax response elements (TREs) (Chan et al. 1994; Simon et al. 1993). *Drosophila* PcG and TrxG proteins are recruited to chromatin by targeting these PREs and TREs, which are *cis*-regulatory DNA elements essential for the regulation of several hundred developmental genes beyond Hox genes. The PcG and TrxG proteins are able to regulate their target genes in a complex and dynamic manner, modifying local chromatin depending on the state of the promoters and maintaining active (TrxG) or repressive (PcG) states. (reviewed in Kassis and Brown, 2013; Geisler and Paro 2015; Grossniklaus and Paro 2014; Steffen and Ringrose, 2014; Ringrose 2017; Schuettengruber et al. 2017). Many PcG/TrxG binding sites give rise to non-coding transcripts (reviewed in Hekimoglu and Ringrose 2009 and Ringrose 2017). For instance, forward and reverse non-coding transcription has been detected from the *Drosophila melanogaster vestigial* (*vg*) PRE/TRE, which switches the status of the element between silencing (induced by transcription from the forward strand) and activation (induced by transcription from the reverse strand). Moreover, the non-coding transcripts from the reverse strand are able to bind to the PRC2 in vivo*,* inhibiting its enzymatic activity (Herzog et al. 2014). Additionally, since the initial discovery of lncRNA *Xist* targeting PcG to the inactive X chromosome in mammals (Plath et al. 2003), several lncRNAs in flies and mammals have been described to participate, not only in PcG-dependent silencing, but also in gene activation via disruption of PcG silencing or physical interaction with TrxG components (Geisler and Paro 2015; Schuettengruber et al. 2017). Altogether, the analyses of non-coding-mediated regulation of PcG and TrxG suggest that non-coding transcripts may be required to destabilize stable active and silent chromatin states, and to recruit or evict components of the PcG and TrxG complexes depending on their transcription rate (Ringrose 2017).

### LncRNAs modulating gene expression

#### LncRNAs transcribed from active enhancers (eRNAs)

Transcription has been observed from multiple active enhancers in mammals (Andersson et al. 2014; Arner et al. 2015; De Santa et al. 2010; Kim et al. 2010), *Drosophila* (Henriques et al. 2018; Meers et al. 2018) and *Caenorhabditis elegans* (Chen et al. 2013). Although these enhancer RNAs (eRNAs) are not transcribed from all enhancer regions, a correlation has been observed between enhancer activity and the transcription of eRNAs both in mammals and flies (Hah et al. 2013; Mikhaylichenko et al. 2018). A growing number of studies demonstrate that specific eRNAs are required to properly activate the expression of their target genes (Ivaldi et al. 2018; Lai et al. 2013; Lam et al. 2013; Li et al. 2013; Rahnamoun et al. 2018; Schaukowitch et al. 2014; Tsai et al. 2018). In mammals, eRNAs have been associated with regulation of transcription through different mechanisms including: interaction and enhancement of the activity of chromatin regulators, like the acetyltransferase CREB binding protein(CBP), PRC2, MLL1 or CTCF; influencing enhancer-promoter looping or altering RNA polymerase II elongation by interaction with proteins that either induce or inhibit elongation (reviewed in De Lara et al. 2019). However, as with the other types of lncRNAs, further studies are required to distinguish the eRNAs that actually play an active role in enhancer activity from those that might just be transcriptional noise arising from the presence of the RNA polymerase machinery. Although few eRNAs have been functionally characterised in flies, identification of general properties of eRNAs in *Drosophila* shows that eRNAs in flies share many characteristics with mammalian eRNAs, for instance, directionality, low abundance, correlation between expression and enhancer activity, or the presence of promoter-like motifs like INR motif (Mikhaylichenko et al. 2018).

### LncRNAs acting at post-transcriptional level

#### LncRNAs as a source of miRNAs

MicroRNAs (miRNAs) are small non-coding transcripts (about 22 nucleotides) that play a major role in the post-transcriptional regulation of gene expression. In most cases, miRNAs are derived from the introns or exons of larger protein-coding or non-coding genes. In *Drosophila*, one of these non-coding transcripts, *iab-8*, is transcribed primarily from the posterior central nervous system, beginning in early development (Bender 2008). It spans over 90 kb and is both spliced and polyadenylated (Bender 2008; Garaulet et al. 2014). Once transcribed, *iab-8* is processed into three miRNAs that altogether are called *miR-iab-8*, which are encoded within its intronic sequence. These miRNAs are known to target and downregulate the homeotic genes *abd-A* and *Ubx*, as well as their cofactors *hth* and *exd* (Garaulet et al. 2014; Gummalla et al. 2012). The consequence of the loss of *iab-8* is male and female sterility caused by the increase in the level of the transcripts targeted by miR-iab-8 that is thought to elicit a defective innervation of the abdominal and/or reproductive tract muscles of the fly (Maeda et al. 2018). In mammals, several lncRNAs have been described as precursors of miRNAs, although none have been found to target the Hox genes. For instance, the maternally imprinted *H19* gene encodes one of the first lncRNAs described, which is a known precursor of *miR-675* (Cai and Cullen 2007). *H19* is highly transcribed in fetal tissues, where it is found to be processed into *miR-675*, which limits placental growth by targeting, among others, growth promoting *Igf1r* (Keniry et al. 2012). In parallel, *H19* is also expressed in the adult skeletal muscle of humans and mice, where, instead of being processed into *miR-675*, *H19* acts as a molecular sponge for the let-7 family of miRNAs (Kallen et al. 2013; Onyango and Feinberg 2011).

Another lncRNA that is processed into smaller RNAs is *acal*, which was described by Riesgo-Escovar and colleagues in 2015 (Ríos-Barrera et al. 2015). *acal* is one of the few *Drosophila* lncRNAs showing sequence conservation. In particular, a 296 nt-long fragment is 80% sequence identical in *Drosophila melanogaster* and *Drosophila bipectinata*. Also, a similar-sized lncRNA is found in humans, showing a considerable 48% sequence identity to *Drosophila acal* (Murillo-Maldonado and Riesgo-Escovar 2019). Mutations in *acal* are embryonic lethal and result in defects in dorsal closure, a JNK-dependent process that is essential for *Drosophila* embryogenesis. It was found that *acal*, through the regulation of two JNK modulators, *Connector of kinase to AP1* (*Cka*) and *anterior open* (*aop*), is able to modulate JNK activity (Ríos-Barrera et al. 2015). Remarkably, *acal* is transcribed from a mono-exonic gene into a 2.3-kb long transcript that, throughout the life cycle of the fly, particularly during pupal stages, is processed into smaller transcripts spanning from 50 to 120 nucleotides. The function of these small RNAs is yet to be investigated, but the differences in size with respect to the ~ 22 nucleotide miRNAs indicate that processed *acal* does not act as a typical miRNA (Ríos-Barrera et al. 2015).

#### LncRNAs regulating isoform usage

We recently identified *blistered antisense* (*bsAS*) as a natural antisense transcript of the *blistered* (*bs)* gene involved in the regulation of *bs* isoform usage in flies (Pérez-Lluch et al. 2020). The *bs* gene encodes the *Drosophila* serum response factor (DSRF) and is a well characterised gene required for wing development and formation (Fristrom et al. 1994; Montagne et al. 1996; Roch et al. 1998). We have found that the usage of *bs* isoforms is regulated in a tissue-specific manner by the expression of the *bsAS*. Transcription of *bsAS* occurs specifically in wing intervein regions and impairs the expression of the long isoforms of *bs*, thereby promoting the relative expression of the short isoform. Overexpression of the long isoform in *bsAS* mutants induces the formation of extra vein tissue in adult wings. The regulation of *bs* isoform usage is based on the formation of a genomic loop between *bs* and *bsAS* promoters that impairs transcription of the long isoform and potentiates short isoform presence. This regulatory mechanism is totally independent of the presence of the *bsAs* transcript, as *bsAS* overexpression does not affect *bs* transcription.

A growing number of lncRNAs has been linked to the modulation of alternative splicing in mammals (reviewed in Romero-Barrios et al. 2018). For example, a natural antisense transcript regulates Zeb2/Sip1 expression during epithelial-mesenchymal transition in mammalian cells by preventing splicing of the Zeb2 5′-UTR (Beltran et al. 2008). An evolutionarily conserved nuclear antisense lncRNA, generated from the human fibroblast growth factor receptor 2 (*FGFR2)* locus, promotes epithelial-specific alternative splicing of *FGFR2* (Gonzalez et al. 2015)*.* This lncRNA impairs the binding of a repressive chromatin-splicing adaptor complex important for mesenchymal-specific splicing, by recruiting PcG proteins and the histone demethylase KDM2a. More recently, Singer and colleagues (Singer et al. 2019) characterised *Paupar,* a lncRNA that interacts with SR proteins to promote the alternative splicing of PAX6 in pancreatic glucagon-producing α cells and computational analysis of hepatocellular carcinoma RNA-Seq samples predicted hundreds of splicing-related lncRNAs (Wang et al. 2020).

### Other mechanisms of action of lncRNAs

#### LncRNAs encoding small functional peptides

By definition, lncRNAs lack protein coding potential. Nevertheless, roughly 98% of the annotated lncRNAs in humans, mice and flies contain small open reading frames (smORFs) of 10 to 100 codons that may code for peptides (Couso and Patraquim 2017). The putative function of these peptides is, however, often neglected and the genes that encode them remain listed as non-coding. Translation of smORFs is observed in many eukaryotes (Andrews and Rothnagel 2014; Couso and Patraquim 2017), but examples of small functional peptides have been described primarily in humans (Anderson et al. 2015; D’Lima et al. 2017; Huang et al. 2017; Nelson et al. 2016; Slavoff et al. 2014; van Heesch et al. 2019) and insects (Galindo et al. 2007; Kondo et al. 2007; Magny et al. 2013). In *Drosophila*, the *tarsal-less* (*tal*) gene, previously classified as non-coding, encodes for a polycistronic mRNA that is translated into 4 small peptides of 11 amino acids. One of these peptides actively participates in leg development at the larval stage by regulating gene expression and tissue folding (Galindo et al. 2007) and at the pupal stage by modulating Notch signalling (Pueyo and Couso 2011). Moreover, the presence of similar smORFs in *tal* homologues across different species of insects suggests the presence of a conserved family of functional peptides (Galindo et al. 2007).

Ribosome profiling techniques (Ribo-seq), which specifically identify ribosome-bound transcripts, have corroborated that a fraction of lncRNAs have a strong affinity for ribosomes (Aspden et al. 2014; Bazzini et al. 2014; Carlevaro-Fita et al. 2016; Ingolia et al. 2011; Ruiz-Orera et al. 2014; van Heesch et al. 2014). However, the association with ribosomes does not necessarily imply that these lncRNAs are actively translated, since lncRNAs are known to regulate the translation of mRNAs through ribosome binding (Carrieri et al. 2012; Hansji et al. 2016; Liu et al. 2019; Yoon et al. 2012). To overcome this limitation, further studies on ribosome-bound lncRNAs should be taken: (1) to confirm whether they are translated and (2) to test the functionality of the translated smORFs. While peptide tagging or in vitro translation assays can be used to identify the coding potential of smORFs (Galindo et al. 2007; Pueyo and Couso 2011; van Heesch et al. 2019), the generation of knock-out mutants or the inhibition of the lncRNA transcription or translation should be considered to study their functionality (Anderson et al. 2015; Pueyo and Couso 2011).

The increasing number of functional smORFs encoded by genes annotated as lncRNAs challenges the current definition of lncRNAs. The fact that almost the totality of annotated lncRNAs present at least one predicted smORF within its sequence makes it impossible to rule them out just because of the smORF presence. However, to our understanding, the lncRNA status of those genes encoding for functionally validated smORFs should be revised or, on the contrary, the definition of lncRNA should be revised to include the genes encoding for functional smORFs.

## Expression of lncRNAs in development

**Fig. 2:**
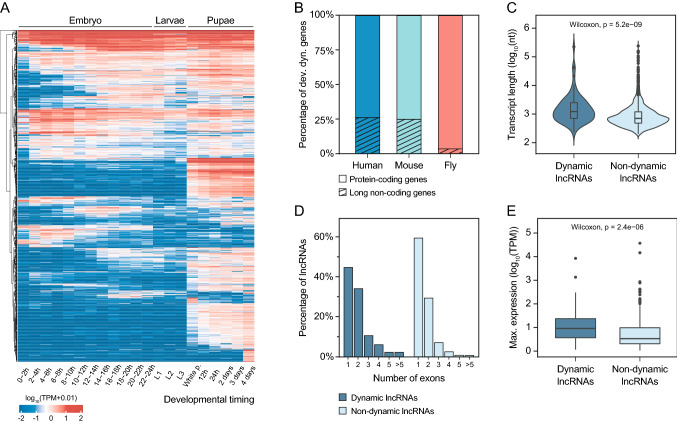
Properties of *Drosophila* lncRNAs in development. **a** Heatmap showing the expression of all the annotated lncRNAs in *Drosophila* RNA-Seq samples from different developmental stages. **b** Percentage of developmentally dynamic genes corresponding to protein-coding and lncRNA genes in humans, mice and flies. Identification of developmentally dynamic genes was performed using edgeR on developmental RNA-Seq samples from Pérez-Lluch et al. (2020). **c** Violin plot showing the distribution of *Drosophila* dynamic and non-dynamic lncRNAs according to the length of their longest transcript. **d** Distribution of *Drosophila* dynamic and non-dynamic lncRNAs according to the number of exons in their longest transcript. **e** Boxplot showing the maximum expression across tissue development (expressed in log_10_TPMs) of dynamic and non-dynamic lncRNAs in *Drosophila*

The first evidence of the involvement of mammalian lncRNAs in development came from high-throughput expression analyses of different tissues (Grote et al. 2013). Cell-type and tissue specificities have been described for many lncRNAs and differential expression of lncRNAs has been reported in in vitro models of haematopoiesis, suggesting that they could have a role in the regulation of cell fate decisions (Briggs et al. 2015; Constanty and Shkumatava 2021; Perry and Ulitsky 2016; Schwarzer et al. 2017). Although most lncRNAs are still uncharacterised, a wide variety of functional activities have been associated with lncRNAs involved in development, such as the regulation of chromatin and DNA interactions, modulation of transcription factors, roles in mRNA stability and processing, and involvement in protein stability and function. Thus, an increasing number of human and mouse lncRNAs are being implicated as key regulators in a variety of cellular processes including proliferation, apoptosis and responses to stress. In agreement with observations in mammals, analyses based on the modENCODE RNA-Seq data from whole *Drosophila* animals have shown that a substantial number of lncRNAs are differentially expressed during development (referred to as developmentally dynamic lncRNAs), although some of the lncRNAs characterised were very lowly expressed (Chen et al. 2016; Brown et al. 2014; Lee et al. 2019; Li et al. 2019). Figure 2a shows the expression changes of the updated list of annotated lncRNAs in *Drosophila* (FlyBase r6.39, 2,545 lncRNAs) across fly development, using the modENCODE RNA-Seq data. Although different profiles of expression can be observed, a huge proportion of lncRNAs is upregulated towards the end of development, as previously reported (Graveley et al. 2011). Indeed, large changes of expression are detected for many genes specifically at the entrance of metamorphosis.

The expression patterns of developmentally dynamic lncRNAs in *Drosophila* are more restricted than those of protein-coding genes. Brown and colleagues reported that, on average, lncRNAs are expressed in a smaller number of stages and tissues compared to protein-coding genes (Brown et al. 2014). Remarkably, similarly restricted expression patterns have been reported for lncRNAs in humans and other mammals (Briggs et al. 2015; Constanty and Shkumatava 2021; Perry and Ulitsky 2016). Most studies characterising lncRNAs expression during development, either in *Drosophila* or in mammals, have been carried out using whole animals, which could be an important constraint considering the high level of tissue specificity that lncRNAs display. Interestingly, a recent publication from Kaessmann’s group systematically described developmentally dynamic lncRNAs across several organs during mammalian development (Sarropoulos et al. 2019). After analysing the RNA-Seq data from seven species, the authors identified developmentally dynamic genes that displayed changes in expression during the development of mammalian organs, showing that the fraction of lncRNAs among this group of genes was substantially low considering the total proportion of lncRNAs in the human and mouse genomes (Sarropoulos et al. 2019). We took advantage of a previously published RNA-Seq data set from our group (Pérez-Lluch et al. 2020) containing the expression values for three tissues (eye, leg and wing) in three developmental stages (third instar larvae, early pupae and late pupae) to identify developmentally dynamic *Drosophila* genes, including lncRNA genes. We observed that the proportion of developmentally dynamic genes corresponding to lncRNAs is much lower in *Drosophila* (4%) than humans and mice (~ 25%), which correlates with the lower number of annotated lncRNAs in flies (Fig. 2b).

We observed that the proportion of lncRNAs within developmentally dynamic genes in flies was lower (3.3%) than that of protein-coding genes (96.7%), a trend stronger than that observed in mammals, in which lncRNAs account for ~ 25% of developmentally dynamic genes (Fig. 2b). Sarropoulos and colleagues found some traits associated with developmentally dynamic lncRNAs. For example, the developmentally dynamic lncRNAs have a higher and broader expression than non-dynamic lncRNAs, they are in closer proximity to protein-coding genes, the transcripts are longer and they contain more exons than non-dynamic lncRNAs. To further characterise dynamic lncRNAs in *Drosophila,* and to compare them with mammalian ones, we analysed the lncRNA length, number of exons and level of expression during tissue development. Developmentally dynamic lncRNAs are longer (Fig. 2c, Mann–Whitney–Wilcoxon test *p*-value = 1.9e−9), contain more exons (Fig. 2d), and generally show higher expression across tissues during development than non-dynamic lncRNAs (Fig. 2e, Mann–Whitney–Wilcoxon test *p*-value = 2.4e−6). Our results indicate, therefore, that the properties identified previously for the mammalian lncRNAs with dynamic expression during organ development are conserved in *Drosophila* developmentally dynamic lncRNAs. Although it is difficult to identify conservation of lncRNAs in different species, the fact that their properties are conserved suggests that some of their roles in development could be conserved, as well.

## Conservation

LncRNA sequences are generally not conserved across different species, which severely hinders the identification of conserved lncRNAs that are likely to be functional. While protein-coding genes are constrained by a strong selective pressure to maintain their reading frame and codon synonymy, lncRNAs do not seem to depend on their sequence to perform their function, leading to their rapid evolution and sequence degeneration. Nevertheless, a few examples of lncRNAs whose sequence is conserved between different species of *Drosophila* have been described. This is the case of the previously discussed lncRNA *acal* or the *yellow-achaete intergenic RNA* (*yar*), which is a lncRNA involved in *Drosophila* sleep regulation. Several motifs ranging from 40 to 111 bp located in the TSS, the exons and the 3′-end of *yar* genomic sequence are conserved in different *Drosophila* species separated by as much as 40–60 million years of evolution (Soshnev et al. 2011). However, it is not possible to find sequence similarity for most lncRNAs, thus, other types of conservation analysis are often used to discover orthologous lncRNAs in different organisms.

Synteny, the positional conservation of neighbouring genes across different species, has emerged as a valuable in identifying orthologous non-coding genes (Bryzghalov et al. 2021; Herrera-Úbeda et al. 2019; Pegueroles et al. 2019; Rolland et al. 2019). This analysis relies on the presence of orthologous genes located in the same order in the linear genome of different species. Syntenic conservation of the region surrounding the lncRNA locus could be an indicator of lncRNA orthology. However, the presence of a lncRNA conserved by synteny in different species does not necessarily imply orthology. Particularly, the presence of large intergenic regions containing multiple lncRNAs increases the rate of false positives (Young et al. 2012). In addition, since the analysis of synteny depends on the presence of orthologous genes, it works better in evolutionarily closer species and becomes less useful as the evolutionary distance increases between the species being compared. Around 60% of protein-coding genes in *Drosophila melanogaster* have human homologues (Wangler et al. 2015), which is often not enough to find orthologous lncRNA genes consistently by the analysis of synteny. Nevertheless, the number of syntenic lincRNAs found in flies and mice is significantly higher than expected by chance, suggesting that a subset of those could be actual orthologs (Young et al. 2012), paving the way for further studies of lncRNAs in *Drosophila*.

Despite lacking sequence conservation, smaller regions of homology among different species have been observed for lncRNAs (Hezroni et al. 2015; Quinn et al. 2016; Ulitsky et al. 2011). These microhomologous regions are thought to correspond to functional elements that are essential for the function of the lncRNA, such as RNA-binding protein motifs or miRNA-binding sites. Recent studies have used a novel approach to identify orthologous lncRNAs based on the identification of these regions of microhomology. It is important to note that RNA-binding protein motifs or miRNA-binding sites are very short (between 4 and 12 nucleotides) and individual matches between different species can be found purely by chance (Bartel 2018). An interesting approach to bypass the rate of false positive hits is the addition of order to these elements (Ross et al. 2021). In this way, not only the presence of these motifs is considered, but also the order in which they are found in putatively orthologous lncRNAs. Although this method has not been tested for distantly related species, finding small regions of homology should be more achievable than finding orthologous lncRNAs using the current methods based on whole-sequence similarity or secondary structure predictions.

Another type of conservation analysis is the study of lncRNA secondary structures, which are thought to be more conserved than the primary sequence (Graf and Kretz 2020; Smith et al. 2013). Unfortunately, the currently available secondary structure predicting tools are not very accurate. Most of these programmes use a minimum free energy algorithm, which predicts the optimal secondary structure that requires the minimum energy to fold. However, features such as the presence of RNA-binding motifs, which should be located outside of the main structure to be properly recognised by their binding proteins, are not taken into consideration. Although it could be a reliable alternative for confirming the orthologous genes identified by other methods, the current secondary structure prediction tools do not seem accurate enough to consider the RNA folding form as the primary source to identify orthologous lncRNAs.

Another approach to identify potential lncRNAs orthologous is through the analysis of their pattern of binding to protein coding genes. If lncRNAs from different species bind to the same orthologous protein coding genes, they may exert a similar function. Methods have been developed to estimate the binding propensity of protein-RNA pairs in silico (Agostini et al. 2013; Armaos et al. 2021; Bellucci et al. 2011). However, to date, this approach has not been tested on a large scale to identify ortholog lncRNAs. To date, no reliable methods exist to systematically establish conservation among lncRNAs in evolutionarily distant species like flies and humans. However, the huge amount of effort made in that direction and the increasing number of annotated transcripts that will emerge in the coming years, hint to a promising perspective regarding lncRNA orthology. The fact that many functions and features associated with lncRNAs are conserved in *Drosophila* reinforces its extraordinary potential as a model organism to functionally characterise and model lncRNAs.

Finally, the characterisation of genomes across the tree of life will provide an incredible amount of data to perform comparative analyses. Advances in sequencing technologies that enable the identification of complete genomes have led to the emergence of the Earth BioGenome Project, an international collaboration that aims to sequence, catalogue and characterise the genomes of all eukaryotes on Earth (Lewin et al. 2018). One of the outcomes of this project is the production of new knowledge on the organisation and evolution of genomes, which could also have a major impact on the field of lncRNAs.

## Data Availability

Not applicable.
